# Three‐Discipline Collaborative Radiation Therapy (3DCRT) debate: Medical physics should focus less on geometric precision and more on biological outcomes

**DOI:** 10.1002/acm2.70636

**Published:** 2026-05-31

**Authors:** François de Kermenguy, Susannah G. Ellsworth, Andrea Facciabene, Marco Durante, Roberto Pacelli, Joel St‐Aubin, Dongxu Wang

**Affiliations:** ^1^ Department of Radiation Oncology Brigham and Women's Hospital, Dana‐Farber Cancer Institute, and Harvard Medical School Boston Massachusetts USA; ^2^ Department of Radiation Oncology University of Pittsburgh Medical Center Pittsburgh Pennsylvania USA; ^3^ Department of Radiation Oncology University of Pennsylvania Philadelphia Pennsylvania USA; ^4^ Ovarian Cancer Research Center University of Pennsylvania Philadelphia Pennsylvania USA; ^5^ Biophysics Department GSI Helmholtzzentrum für Schwerionenforschung Darmstadt Germany; ^6^ Institute for Condensed Matter Physics Technische Universität Darmstadt Darmstadt Germany; ^7^ Department of Physics “Ettore Pancini” University Federico II Naples Italy; ^8^ Department of Advanced Biomedical Sciences University Federico II Naples Italy; ^9^ Department of Radiation Oncology University of Iowa Health Care Iowa City Iowa USA; ^10^ Department of Medical Physics Memorial Sloan Kettering Cancer Center New York New York USA

## 1 THREE‐DISCIPLINE COLLABORATIVE RADIATION THERAPY (3DCRT) DEBATE SERIES

Radiation oncology is a highly multidisciplinary medical specialty, drawing significantly from three scientific disciplines—medicine, physics, and biology. As a result, discussion of controversies or changes in practice within radiation oncology involves input from all three disciplines. This journal has adopted this “team‐science” approach to debate articles in the “parallel‐opposed” editorials. This article is part of the long‐running debate series entitled “three‐discipline collaborative radiation therapy (3DCRT)”. In the “3DCRT” debate, each debate team has included three multidisciplinary team members, with the hope that this format would both engage the readership and foster further collaboration in the science and clinical practice of radiation oncology.

## 2 INTRODUCTION

Medical physicists contribute to radiation therapy of cancer through their work in radiation dosimetry, radiation delivery, and quantitative modeling of radiobiology. The past decades have seen remarkable progress in the precision of radiation delivery. At the same time, clinical oncology has progressed significantly through the better understanding of cancer biology. In this context, we face the question whether medical physicists should now prioritize radiobiology‐driven work, instead of continuing to emphasize the traditional, incremental improvement in spatial accuracy of radiation delivery. The debate proposition in this article states: “*Medical physics should focus less on geometric precision and more on biological outcomes*.”

Arguing for this proposition are Dr. François de Kermenguy, Dr. Susannah G. Ellsworth, Dr. Andrea Facciabene.

Dr. de Kermenguy is a postdoctoral research fellow and incoming resident of Medical Physics at Harvard Medical School and in the Department of Radiation Oncology at Brigham and Women's Hospital and the Dana‐Farber Cancer Institute. His PhD research merged physics with mathematical oncology to investigate the radiation dose delivered to lymphocytes during treatment and ultimately enable radiotherapy/immunotherapy combinations. His postdoctoral work focuses on the development and optimization of a new dual‐energy imager for patient set‐up verification in radiotherapy.

Dr. Ellsworth is a clinical associate professor in the Department of Radiation Oncology at the University of Pittsburgh. Clinically, she specializes in gastrointestinal cancers with an interest in pancreatic and colorectal malignancies. Her research interests focus on radiation effects on the immune system and novel treatment approaches for pancreatic cancer.

Dr. Facciabene is a research associate professor in the Department of Radiation Oncology and the Ovarian Cancer Research Center at the University of Pennsylvania. He leads a translational research laboratory dedicated to advancing tumor immunology and immunotherapy. His pioneering work includes developing novel preclinical immunotherapies and vaccination strategies—such as utilizing mitochondria isolated from tumors—as well as exploring how the gut microbiome regulates immune surveillance and the tumor microenvironment.

Arguing against this proposition are Dr. Marco Durante, Dr. Roberto Pacelli, and Dr. Joel St‐Aubin.

Dr. Durante is the director of the Biophysics Department at the GSI Helmholtzzentrum für Schwerionenforschung in Darmstadt, Germany, and full professor of Physics at the Technische Universität Darmstadt and part‐time at the University of Naples Federico II in Italy. Dr. Durante's research primarily addresses radiation biophysics, the optimization of charged particle therapy, and space radiation protection.

Dr. Pacelli is a full professor of Diagnostic Imaging and Radiotherapy in the Department of Advanced Biomedical Sciences at the University of Naples Federico II in Naples, Italy. His clinical and academic focus lies in radiation oncology, where he has published on mathematical modeling and analysis of radiation effect in various disease types.

Dr. St‐Aubin is a clinical associate professor and is the physics director of stereotactic radiosurgery in the Department of Radiation Oncology at the University of Iowa Health Care, in Iowa City, USA. His clinical and research expertise centers on advanced dosimetric modeling and the clinical implementation of MRI‐guided adaptive radiotherapy. He is particularly focused on optimizing treatment planning delivery times using artificial intelligence for online adaptive radiotherapy.

## 3 OPENING STATEMENTS

### 3.1 Opening statement for the proposition

The historical trajectory of radiation oncology (RT) has been defined by a steady focus on geometric precision, prioritizing conformality and margin reduction through technologies like intensity‐modulated RT, image‐guided RT, and particle therapy. However, this relentless pursuit of dosimetric perfection risks becoming what Richard Feynman termed “cargo cult science”: performing the rituals of scientific rigor without achieving the requisite intellectual substance.^1^, ^2^ This evolution of RT has been mostly driven by reliance on the linear quadratic (LQ) model,^3^ with the assumption that treatment planning with better target coverage and organ at risk (OAR) sparing will inevitably result in improvements in disease control and normal tissue toxicity.

Like the unfortunate travelers of Greek mythology who found themselves either stretched out or chopped down to fit into the fatal bed of Procrustes, radiation oncologists are currently forced into rigid treatment paradigms that apply the same regimen in all patients (albeit with millimeter‐level precision!), regardless of significant inter‐patient heterogeneities in tumor and normal tissue radiosensitivity.^4^ Therefore, current RT practice relies on Biological Effective Dose (BED) or Equivalent Dose in 2 Gy fractions (EQD2) matching to compare and rationalize different fractionation schedules under the assumption of biological equivalence.^3^, ^5^ While such approaches may be useful for normalizing predicted direct tumor cell kill or late normal tissue toxicity within the constraints of the LQ framework, they implicitly assume that dose–response relationships are monotonic, context‐independent, and dominated by clonogenic survival.

The reality, however, is far more complex. By focusing solely on the loss of cellular proliferation capacity induced by DNA damage, the LQ model neglects “passenger” effects of RT that are essential for disease eradication, such as death mechanisms and immune modulation.^6^ In particular, immune‐mediated effects of radiotherapy, including antigen release, antigen‐presenting cell activation, lymphocyte depletion, vascular modulation, myeloid reprogramming, and the induction of systemic antitumor responses (abscopal effects), depend strongly on dose per fraction, spatial dose distribution, treatment volume, and timing relative to systemic therapies, often in a non‐linear and non‐monotonic way.^7^, ^8^ As a result, fractionation schedules that are formally equivalent by BED may yield profoundly different biological and clinical outcomes, particularly in combination with immunotherapy.^9^, ^10^ An additional and largely unaccounted determinant of radiotherapy response in this context is the gut microbiome, which exerts systemic effects on antitumor immunity and host metabolism. Radiotherapy can reshape the gut microbiota,^11^, ^12^ while baseline microbial composition and microbiota‐derived metabolites modulate antigen presentation, T‐cell priming, myeloid cell function, and responsiveness to immunotherapy.^13^, ^14^ These microbiome–immune interactions are not captured by physical dose metrics or BED‐based equivalence and may be differentially influenced by dose per fraction, irradiated volume, and treatment timing. Consequently, dosimetrically equivalent regimens may induce distinct systemic immune and metabolic states through microbiome‐mediated mechanisms, further undermining the assumption that biological response can be inferred from dose alone.^11^, ^15^, ^16^, ^17^

Beyond the biological limitations, the race for nanometric radiotherapy presents societal and economic challenges that do not fit with a value‐based radiotherapy pragmatic approach.^18^, ^19^, ^20^ The field must differentiate between technological novelty and genuine clinical value to ensure the sustainability of healthcare financing. Unchecked investment in technologies that solely improve dosimetry without proven benefits to disease control or toxicity reduction wastes limited resources and hampers equitable patient access.^21^ Moreover, in an era of environmental crisis^22^, ^23^ and geopolitical instability, the reliance on complex hardware‐heavy technologies reduces the resilience of oncology departments by inflating human resource requirements^24^ and introducing multiple points of failure within both the clinical workflow and supply chain.^25^

To address these challenges, the focus of radiation oncology must pivot from technological escalation focused on geometric precision to biologically guided planning and delivery of RT. In addition to new ways to deliver radiation (such as low‐dose irradiation,^11^, ^26^, ^27^ spatially fractionated radiation therapy^28^ or FLASH therapy^29^, ^30^), resources should be redirected toward unraveling the mechanisms of radioresistance, cell death pathways, inter‐individual variations in radiation response (across both malignant and healthy tissues), radiation‐driven immune modulation, and microbiome‐mediated effects. While the tumor control probability is likely determined not purely by dose deposited but also by the balance of immunostimulatory and immunosuppressive effects of local RT, the formal integration of immuno‐oncologic insights into radiotherapy planning remains elusive. Addressing this gap is essential to resolving the inconsistent results observed in recent immuno‐radiotherapy trials.^31^, ^32^ Medical physicists are uniquely positioned to lead this transition, not by merely refining dose distributions, but by applying their expertise to overcome the LQ model and develop new tools and biomarkers^33^, ^34^, ^35^, ^36^, ^37^, ^38^ needed for the next generation of treatment planning systems.^39^ By integrating biological complexity into treatment planning, the field can move beyond the geometric precision paradigm toward a truly personalized approach that prioritizes clinical outcomes over dosimetric aesthetics.

### 3.2 Opening statement against the proposition

Medical physics should not focus less on geometric precision in favor of biological outcomes. Rather, geometric precision is the indispensable foundation upon which any meaningful biologically guided radiotherapy (RT) must be built.

The motion presents a false dichotomy. Nobody these days disputes the importance of tumor biology, immune modulation, the microbiome, or inter‐individual sensitivity. These emerging domains will undoubtedly shape the future of RT.^40^ However, the promise of biologically individualized RT cannot be realized unless radiation is first delivered accurately, reproducibly, and selectively to the intended target.^41^

RT is unique among cancer treatments because it is defined not merely by what is delivered, but by where it is delivered. A given dose has profoundly different consequences depending on its spatial distribution. Immune activation,^42^ lymphocyte depletion,^33^ vascular remodeling,^10^ and normal tissue toxicity^43^ are all strongly dependent on the volume and location of irradiated tissues. Thus, biological outcomes are inherently spatial phenomena. In radiotherapy, geometry governs biology.

To reduce RT to “give a very high dose to the target sparing the normal tissue as much as possible” is naïve. Nevertheless, the emphasis on geometric precision led to major clinical gains. Image‐guided radiotherapy, intensity modulation, stereotactic techniques, adaptive workflows, and margin reduction have enabled dose escalation while reducing toxicity. These advances have translated into improved tumor control and quality of life across multiple disease sites. In localized lung^44^ and prostate^45^ cancer, precision RT confers to the patients the same cure probability of surgery, allowing to successfully treat even patients non‐medically fit for surgery. Sparing salivary glands in head‐and‐neck cancer,^46^ bowel in pelvic irradiation,^47^ heart and lung in breast cancer,^48^ and healthy brain in radiosurgery^49^ are clinically meaningful outcomes made possible by precision.

Particle therapy provides a particularly compelling counterexample to the motion. Its clinical value derives directly from superior geometric selectivity, which translates into measurable biological and clinical benefits.^50^ A typical case is pediatric craniospinal irradiation, where proton therapy significantly reduces integral dose to the heart, lungs, and abdomen, lowering the risk of late toxicity, endocrine dysfunction, and secondary malignancies.^51^ Craniospinal irradiation with protons can also prolong survival in adult patients with leptomeningeal metastasis compared to conventional X‐rays.^52^ In left‐sided breast cancer, proton therapy markedly reduces cardiac dose, with important implications for long‐term cardiovascular morbidity.^53^ In oropharyngeal carcinoma, recent results of a randomized trial in the US show that the high precision of protons reduces toxicity and leads to prolonged patients' survival.^54^

In re‐irradiation, where normal tissue tolerance is already limited, proton therapy often determines whether curative treatment is feasible at all by minimizing additional dose to previously irradiated organs.^55^ Furthermore, reducing the low‐dose bath with charged particles results in lower lymphopenia, directly linking geometric precision to immune preservation.^56^ Carbon ion therapy further illustrates that biological effectiveness is inseparable from spatial accuracy; without precise delivery, its advantages would be lost.^57^

Adaptive radiotherapy (ART) provides an equally compelling example of the benefits of geometric precision. MR‐Linacs are an example of ART technology that provides high‐quality imaging essential for daily dose adaptations and real‐time motion management.^58^ MR‐Linac based ART provides increased geometric targeting accuracy which in turn enables the reduction of treatment uncertainty margins. The improved targeting accuracy and smaller treatment margins associated with MR‐Linac ART have shown significant reductions in grade ≥2 genitourinary toxic effects for stereotactic body radiotherapy prostate cancer treatments.^59^

Critiques of current radiobiological models, including the limitations of the linear‐quadratic framework, are valid. However, they do not justify de‐emphasizing precision. Instead, they reinforce the need to integrate improved biological understanding with advanced delivery capabilities, such as MR‐Linac. If treatment planning evolves to incorporate genomic radiosensitivity, immune status, or microbiome‐derived biomarkers, these insights must be translated into spatially resolved dose prescriptions. Such strategies, such as dose painting,^60^ require more precise targeting, not less. Indeed, the more sophisticated our biological understanding becomes, the more demanding geometric requirements will be. If radioresistant subregions within tumors are to be selectively escalated, or if immune‐sensitive structures such as lymph nodes, bone marrow, or circulating blood are to be spared, then millimetric accuracy becomes essential.

Deprioritizing geometric precision risks unintended harm. Broader margins or less conformal treatments could increase toxicity, compromise quality of life, and ultimately undermine clinical outcomes. A shift away from precision may paradoxically impair the very biological effects the motion seeks to prioritize.

Economic and societal concerns about advanced technologies should be framed in terms of value rather than complexity. Technologies that reduce severe toxicity, prevent long‐term complications, and enable curative treatment in challenging scenarios are not luxuries; they are high‐value interventions. Moreover, precision technologies often become more efficient and accessible over time.

The future of medical physics is therefore not a choice between geometry and biology. Biology informs what should be done; geometry determines whether it can be done safely and effectively. Geometric precision is not the obstacle to biologically guided radiotherapy: it is the platform that makes it possible.

## 4 REBUTTALS

### 4.1 Rebuttal, for the proposition

Geometric precision has undeniably improved the safety of radiotherapy. However, the central question of this debate is not whether precision matters, but whether further refinements in geometric delivery continue to translate into meaningful clinical benefit. On this point, the evidence is far less convincing. While toxicity management is crucial, the primary purpose of RT is to treat lesions. Thus far, outside the specific subset of patients eligible for ablative dose escalation via SBRT,^61^ superior dose precision has not always translated into significant improvements in OS or PFS.^62^

Our colleagues highlighted proton therapy as a compelling counterexample to the motion. However, several large recent phase III studies in patients with prostate (PARTIQoL^63^), breast (RadComp^53^), and head and neck cancers (TORPEdO^64^) have not demonstrated consistent improvements in survival and have shown at best modest or context‐dependent reductions in toxicity compared to modern photon techniques.^62^ We acknowledge that a recent landmark phase III trial in patients with oropharyngeal cancer reported a significant OS improvement for proton versus photon therapy.^54^ However, the study clearly highlights reduced lymphopenia and immune–oncology–microbiome axis alterations in the proton arm as “potential mechanisms underlying the improvement in overall survival”. These results challenge the assumption that superior geometric conformity necessarily improves outcomes, indicating that geometry alone is not sufficient to consistently drive meaningful clinical benefit.

It is also argued that adaptive radiotherapy (ART) enables further reduction of PTV margins. However, investing additional resources into perfecting physical delivery is unlikely to yield substantial additional clinical benefit within the current “one‐size‐fits‐all” dosimetric paradigm.^65^ Such a framework fails to account for patient‐specific patterns of tumor infiltration and the profound inter‐individual variability in both tumor and normal tissue radiation responses.^4^, ^37^, ^66^, ^67^ In this setting, increased geometric precision will simply deliver “biologically imprecise” treatment with greater physical accuracy. Future outcome improvements will therefore likely stem from CTV and dose personalization delineation leveraging advanced imaging and disease infiltration models.

Finally, modern oncology is fundamentally reliant on combination therapies. Protocols optimized purely for cytotoxic effects are rarely optimal for immunological synergy with immune checkpoint blockers and other systemic therapies.^31^ While potentially useful, defining new, fixed dose constraints to spare immune organs remains a “business as usual” approach.^68^ Next‐generation treatment planning should not treat the immune system simply as a passive organ at risk, but as an active lever that can be engaged to synergize with RT in order to enhance therapeutic efficacy.

In conclusion, we contend that while maintaining tight geometric margins is critical for clinical safety, the next major breakthrough in radiotherapy will not come from further submillimetric physical refinements, but rather from biological optimization. First, highly precise techniques such as proton therapy, while effective at improving dose distribution in tissue, have not consistently demonstrated survival benefits in clinical trials. Second, the “geometric race” is fundamentally undermined by a lack of personalization, both in terms of CTV design and dosing: it is futile to perfect the “where” when we remain uncertain about the “what,” the “how much,” and even the “when”.^69^ Third, the mixed clinical outcomes observed in recent immuno‐radiotherapy trials^32^ suggest that merely tweaking current protocols is insufficient to optimize radiotherapy integration with these next‐generation modalities.^31^ Medical physicists have a unique opportunity to embrace the fast‐evolving landscape of modern oncology by applying their expertise to question how radiation dose and delivery technique modulate tumor biology, especially with respect to the antitumor immune response.

### 4.2 Rebuttal, against the proposition

Our colleagues rightly emphasize the limitations of current biological models and the importance of incorporating immune effects, radiosensitivity, and the microbiome into radiotherapy. We agree with this vision. However, their argument rests on the incorrect premise that an emphasis on geometric precision has constrained biological progress. In reality, precision has enabled it.

Many of the biological effects highlighted in support of the motion are themselves critically dependent on dose geometry. Radiation‐induced lymphopenia is strongly influenced by the volume of circulating blood, bone marrow, and lymphoid organs exposed to radiation.^70^ Microbiome perturbation depends on the extent and location of bowel irradiation.^71^ Immune activation and abscopal effects vary with treated volume, dose heterogeneity, and exposure of draining lymph nodes.^72^ These observations do not argue against geometric precision—they demonstrate its central biological relevance.

We agree that equivalent dose regimens (BED or EQD2) can produce different outcomes, but this does not justify deprioritizing the most controllable and quantifiable aspect of radiotherapy. Geometry remains the foundation upon which biological hypotheses can be tested, validated, and ultimately implemented in clinical practice.

Furthermore, many of the proposed biologically driven strategies demand even greater precision. Particle therapy and ART both illustrate this point. The ability of protons to reduce integral dose and spare immune‐relevant structures depends entirely on accurate beam placement and range control.^73^ The biological promise of carbon ion therapy is inseparable from its spatial precision.^74^ ART enables reduced treatment margins with subsequent reductions in normal tissue toxicities.^75^ Without geometric accuracy, these biological advantages would not translate into clinical benefit.

Finally, characterizing advances in precision as “dosimetric aesthetics” overlooks their demonstrated clinical impact. Reductions in xerostomia, dysphagia, genitourinary toxicity, neurocognitive decline, cardiac toxicity, and secondary malignancies are tangible improvements in patient outcomes, not abstract dosimetric gains.

The appropriate conclusion is not that medical physics should focus less on geometric precision, but that it should leverage precision in service of increasingly sophisticated biological goals. Geometry and biology are fundamentally interdependent.

## CONFLICT OF INTEREST STATEMENT

François de Kermenguy is supported by a research fellowship funded by Varian Medical Systems (Palo Alto, CA, USA), unrelated to this work. Marco Durante is currently president of the Particle Therapy Co‐Operative Group (PTCOG) and is recipient of several research grants from public funding sources. Joel St‐Aubin has industry sponsored grants from Elekta AB (Stockholm, Sweden) unrelated to this work. Dongxu Wang receives licensing fee payment from Ion Beam Applications, S.A. (Louvain‐la‐Neuve, Belgium), unrelated to this work.

## References

[1] Feynman RP . Cargo cult science. Eng Sci. 1974;37(7):10‐13.

[2] Ellsworth SG , Wilke C . Cargo cult radiotherapy: the illusion of precision in advanced technologies. Cureus. 2025;17(2):e79005. 10.7759/cureus.79005 40099053 10.7759/cureus.79005 PMC11911286

[3] McMahon SJ . The linear quadratic model: usage, interpretation and challenges. Phys. Med. Biol. 2018;64(1):01TR01. 10.1088/1361‐6560/aaf26a 10.1088/1361-6560/aaf26a 30523903

[4] Hsu K‐S , M Adileh , M L Martin , et al. Colorectal cancer develops inherent radiosensitivity that can be predicted using patient‐derived organoids. Cancer Res. 2022;82(12):2298‐2312. doi: 10.1158/0008‐5472.CAN‐21‐4128 35472075 10.1158/0008-5472.CAN-21-4128 PMC9390071

[5] Y. Lee , S. L. Auh , Y. Wang , et al. Therapeutic effects of ablative radiation on local tumor require CD8+ T cells: changing strategies for cancer treatment. Blood. 2009;114(3):589‐595. doi: 10.1182/blood‐2009‐02‐206870 19349616 10.1182/blood-2009-02-206870 PMC2713472

[6] S. Demaria , C. Guha , J. Schoenfeld , et al. Radiation dose and fraction in immunotherapy: one‐size regimen does not fit all settings, so how does one choose?. J Immunother Cancer. 2021;9(4):e002038. doi: 10.1136/jitc‐2020‐002038 33827904 10.1136/jitc-2020-002038 PMC8031689

[7] S. C Formenti , S. Demaria . Radiation therapy to convert the tumor into an in situ vaccine. Int J Radiat Oncol Biol Phys. 2012;84(4):879‐880. doi: 10.1016/j.ijrobp.2012.06.020 23078897 10.1016/j.ijrobp.2012.06.020 PMC3811126

[8] G. D Grass , N. Krishna , S. Kim . The immune mechanisms of abscopal effect in radiation therapy. Curr Probl Cancer. 2016;40(1):10‐24. doi: 10.1016/j.currproblcancer.2015.10.003 26612692 10.1016/j.currproblcancer.2015.10.003

[9] C. Vanpouille‐Box , A. Alard , M. J. Aryankalayil , et al. DNA exonuclease Trex1 regulates radiotherapy‐induced tumour immunogenicity. Nat Commun. 2017;8(1):15618. doi: 10.1038/ncomms15618 28598415 10.1038/ncomms15618 PMC5472757

[10] H. E Barker , J. T E. Paget , A. A Khan , K. J Harrington . The tumour microenvironment after radiotherapy: mechanisms of resistance and recurrence. Nat Rev Cancer. 2015;15(7):409‐425. doi: 10.1038/nrc3958 26105538 10.1038/nrc3958 PMC4896389

[11] J. Chen , A. Levy , A.i‐L. Tian , et al. Low‐dose irradiation of the gut improves the efficacy of PD‐L1 blockade in metastatic cancer patients. Cancer Cell. 2025;43(3):361‐379.e10. doi: 10.1016/j.ccell.2025.02.010 40068595 10.1016/j.ccell.2025.02.010 PMC11907695

[12] J. Chen , E. Deutsch , G. Kroemer , L. Galluzzi , L. Zitvogel . The microbiota in radiotherapy‐induced cancer immunosurveillance. Nat Rev Clin Oncol. 2025;22(9):667‐679. doi: 10.1038/s41571‐025‐01052‐8 40659791 10.1038/s41571-025-01052-8

[13] S. P Wiertsema , J. van Bergenhenegouwen , J. Garssen , L. M J. Knippels . The interplay between the gut microbiome and the immune system in the context of infectious diseases throughout life and the role of nutrition in optimizing treatment strategies. Nutrients. 2021;13(3):886. doi: 10.3390/nu13030886 33803407 10.3390/nu13030886 PMC8001875

[14] D. Zheng , T. Liwinski , E. Elinav . Interaction between microbiota and immunity in health and disease. Cell Res. 2020;30(6):492‐506. doi: 10.1038/s41422‐020‐0332‐7 32433595 10.1038/s41422-020-0332-7 PMC7264227

[15] W. Ma , Q. Mao , W. Xia , G. Dong , C. Yu , F. Jiang . Gut microbiota shapes the efficiency of cancer therapy. Front Microbiol. 2019;10:1050. doi: 10.3389/fmicb.2019.01050 31293523 10.3389/fmicb.2019.01050 PMC6604670

[16] V. Gopalakrishnan , C. N. Spencer , L. Nezi , et al. Gut microbiome modulates response to anti–PD‐1 immunotherapy in melanoma patients. Science. 2018;359(6371):97‐103. doi: 10.1126/science.aan4236 29097493 10.1126/science.aan4236 PMC5827966

[17] M. Uribe‐Herranz , S. Rafail , S. Beghi , et al. Gut microbiota modulate dendritic cell antigen presentation and radiotherapy‐induced antitumor immune response. J Clin Invest. 2020;130(1):466‐479. doi: 10.1172/JCI124332 31815742 10.1172/JCI124332 PMC6934221

[18] A call for pragmatism in cancer research. (2018). Nat Rev Clin Oncol, 15(4):1759‐4782. doi: 10.1038/nrclinonc.2018.41 10.1038/nrclinonc.2018.41 29553132

[19] Value‐based radiotherapy: a new chapter of the ESTRO‐HERO project. (2021). Radiother Oncol, 160: 236‐239. doi: 10.1016/j.radonc.2021.05.007 33992629 10.1016/j.radonc.2021.05.007

[20] S. Teckie , S. A McCloskey , M. L Steinberg . Value: a framework for radiation oncology. J Clin Oncol. 2014;32(26):2864‐2870. doi: 10.1200/JCO.2014.55.1150 25113759 10.1200/JCO.2014.55.1150 PMC4152714

[21] S. Teckie , D. Koffler , L. Potters . The resilience of radiation oncology in the COVID era and beyond. Int J Radiat Oncol Biol Phys. 2020;108(2):364‐369. doi: 10.1016/j.ijrobp.2020.06.065 32890514 10.1016/j.ijrobp.2020.06.065 PMC7462879

[22] Green horizons in oncology: a blueprint for environmentally sustainable radiation therapy facilities. Semin Radiat Oncol. 2024;34(4):426‐432. doi: 10.1016/j.semradonc.2024.07.004 39271277 10.1016/j.semradonc.2024.07.004

[23] K. E. Lichter , K. Charbonneau , J. R. Lewy , et al. Quantification of the environmental impact of radiotherapy and associated secondary human health effects: a multi‐institutional retrospective analysis and simulation. Lancet Oncol. 2024;25(6):790‐801. doi: 10.1016/S1470‐2045(24)00148‐7 38821084 10.1016/S1470-2045(24)00148-7

[24] K. Thind , M. Roumeliotis , T. Mann , et al. Increasing demand on human capital and resource utilization in radiation therapy: the past decade. Int J Radiat Oncol Biol Phys. 2022;112(2):457‐462. doi: 10.1016/j.ijrobp.2021.09.020 34543682 10.1016/j.ijrobp.2021.09.020

[25] T. Drew , A. Conway , I. Do , et al. Impact of extreme weather events on radiation oncology practices in Puerto Rico. Cancer Control. 2025;32:10732748251394061. doi: 10.1177/10732748251394061 41414884 10.1177/10732748251394061 PMC12717360

[26] P.‐A. Laurent , L. Shi , L. Bouarroudj , et al. Low‐dose radiotherapy enhances the efficacy of PD‐L1 blockade and induces the abscopal effect. J Immunother Cancer. 2025;13(6):e011487. doi: 10.1136/jitc‐2025‐011487 40588370 10.1136/jitc-2025-011487 PMC12211843

[27] P. Bergeron , M. Dos Santos , L. Sitterle , et al. Non‐homogenous intratumor ionizing radiation doses synergize with PD1 and CXCR2 blockade. Nat Commun. 2024;15(1):8845. doi: 10.1038/s41467‐024‐53015‐9 39397001 10.1038/s41467-024-53015-9 PMC11471822

[28] Y. Prezado , M. Grams , E. Jouglar , et al. Spatially fractionated radiation therapy: a critical review on current status of clinical and preclinical studies and knowledge gaps. Phys. Med. Biol. 2024;69(10):10TR02. doi: 10.1088/1361‐6560/ad4192 10.1088/1361-6560/ad4192 38648789

[29] M. C. Vozenin , P. Montay‐Gruel , P. Tsoutsou , C. L Limoli . Mechanisms, challenges and opportunities for FLASH radiotherapy in cancer. Nat Rev Cancer. 2026; 26 62‐75. doi: 10.1038/s41568‐025‐00878‐9 41136613 10.1038/s41568-025-00878-9 PMC12951891

[30] H. Ni , Z. J. Reitman , W. Zou , et al. FLASH radiation reprograms lipid metabolism and macrophage immunity and sensitizes medulloblastoma to CAR‐T cell therapy. Nature Cancer. 2025;6(3):460‐473. doi: 10.1038/s43018‐025‐00905‐6 39910249 10.1038/s43018-025-00905-6 PMC12244404

[31] L. B Darragh , S. D Karam . Radiation as an immune modulator: mechanisms and implications for combination with immunotherapy. Nat Rev Cancer. 2026; 26: 270‐284. doi: 10.1038/s41568‐025‐00903‐x 41577962 10.1038/s41568-025-00903-x

[32] A. Levy , C. Massard , S. Michiels , E. Deutsch . Innovative, early‐phase clinical trials of drug–radiotherapy combinations. Lancet Oncol. 2025;26(4):e190‐e202. doi: 10.1016/S1470‐2045(24)00664‐8 40179915 10.1016/S1470-2045(24)00664-8

[33] F. de Kermenguy , D. Morel , M. El‐Aichi , D. Barbolosi , E. Deutsch , C. Robert . Radiation‐induced lymphopenia: from mathematical modeling towards mechanistic learning. Int J Radiat Oncol Biol Phys. 2026;124(2):465‐483. doi: 10.1016/j.ijrobp.2025.07.1429 40763901 10.1016/j.ijrobp.2025.07.1429

[34] K. R. Scibilia , K. Gallagher , M. A. Masud , et al. Mathematical oncology: how modeling is transforming clinical decision‐making. Cancer Res. 2025;85(24):4866‐4879. doi: 10.1158/0008‐5472.CAN‐25‐0750 41105675 10.1158/0008-5472.CAN-25-0750 PMC12614879

[35] F. Pradelli , M. Strobl , S. Marzban , et al. 75 years of mathematical oncology. Biorxiv. Preprint, posted January 14, 2026. doi: 10.64898/2026.01.13.699306.

[36] R. Sun , E. J. Limkin , M. Vakalopoulou , et al. A radiomics approach to assess tumour‐infiltrating CD8 cells and response to anti‐PD‐1 or anti‐PD‐L1 immunotherapy: an imaging biomarker, retrospective multicohort study. Lancet Oncol. 2018;19(9):1180‐1191. doi: 10.1016/S1470‐2045(18)30413‐3 30120041 10.1016/S1470-2045(18)30413-3

[37] J. G. Scott , A. Berglund , M. J. Schell , et al. A genome‐based model for adjusting radiotherapy dose (GARD): a retrospective, cohort‐based study. Lancet Oncol. 2017;18(2):202‐211. doi: 10.1016/S1470‐2045(16)30648‐9 27993569 10.1016/S1470-2045(16)30648-9 PMC7771305

[38] F. de Kermenguy , N. Benzazon , P. Maury , et al. LymphoDose: a lymphocyte dose estimation framework—application to brain radiotherapy. Phys Med Biol. 2024;69(10):105009. doi: 10.1088/1361‐6560/ad3c8d 10.1088/1361-6560/ad3c8d 38593817

[39] D. Morel , C. Robert , N. Paragios , V. Grégoire , E. Deutsch . Translational frontiers and clinical opportunities of immunologically‐fitted radiotherapy. Clin Cancer Res. 2024;30(11):2317‐2332. doi: 10.1158/1078‐0432.CCR‐23‐3632 38477824 10.1158/1078-0432.CCR-23-3632 PMC11145173

[40] M. Baumann , M. Krause , J. Overgaard , et al. Radiation oncology in the era of precision medicine. Nat Rev Cancer. 2016;16(4):234‐249. doi: 10.1038/nrc.2016.18 27009394 10.1038/nrc.2016.18

[41] R. Pacelli , M. Caroprese , G. Palma , et al. Technological evolution of radiation treatment: implications for clinical applications. Semin Oncol. 2019;46(3):193‐201. doi: 10.1053/j.seminoncol.2019.07.004 31395286 10.1053/j.seminoncol.2019.07.004

[42] G. Petroni , L. C Cantley , L. Santambrogio , S. C Formenti , L. Galluzzi . Radiotherapy as a tool to elicit clinically actionable signalling pathways in cancer. Nat Rev Clin Oncol. 2022;19(2):114‐131. doi: 10.1038/s41571‐021‐00579‐w 34819622 10.1038/s41571-021-00579-w PMC9004227

[43] D. E Citrin , R. D Timmerman . Effects of radiotherapy in normal tissue. N Engl J Med. 2026;394(10):996‐1009. doi: 10.1056/NEJMra2506017 41780002 10.1056/NEJMra2506017

[44] J. Y. Chang , S. Senan , M. A. Paul , et al. Stereotactic ablative radiotherapy versus lobectomy for operable stage I non‐small‐cell lung cancer: a pooled analysis of two randomised trials. Lancet Oncol. 2015;16(6):630‐637. doi: 10.1016/S1470‐2045(15)70168‐3 25981812 10.1016/S1470-2045(15)70168-3 PMC4489408

[45] L. Hekman , A. Barrett , D. Ross , et al. A systematic review of clinical trials comparing radiation therapy versus radical prostatectomy in prostate cancer. Clin. Genitourin. Cancer. 2024;22(5):102157. doi: 10.1016/j.clgc.2024.102157 39084158 10.1016/j.clgc.2024.102157

[46] P. G. Hawkins , J. Y. Lee , Y. Mao , et al. Sparing all salivary glands with IMRT for head and neck cancer: longitudinal study of patient‐reported xerostomia and head‐and‐neck quality of life. Radiother Oncol. 2018;126(1):68‐74. doi: 10.1016/j.radonc.2017.08.002 28823405 10.1016/j.radonc.2017.08.002

[47] J. Lee , J.‐B. Lin , C.‐S. Weng , S.‐J. Chen , T.‐C. Chen , Y.‐J. Chen . Impact of reduced margin pelvic radiotherapy on gastrointestinal toxicity and outcome in gynecological cancer. Clin Transl Radiat Oncol. 2023;43:100671. doi: 10.1016/j.ctro.2023.100671 37692995 10.1016/j.ctro.2023.100671 PMC10482739

[48] Y. Kim , J. E Bates , H. I Yoon , C. Grassberger . Cardiac radiosensitivity in the era of thoracic chemoradiotherapy and immunotherapy: a scoping review. Lancet Oncol. 2026;27(3):e130‐e140. doi: 10.1016/S1470‐2045(25)00651‐5 41785903 10.1016/S1470-2045(25)00651-5 PMC13492064

[49] R. S. Prabhu , T. Akinyelu , Z. K. Vaslow , et al. Risk factors for progression and toxic effects after preoperative stereotactic radiosurgery for patients with resected brain metastases. JAMA Oncol. 2023;9(8):1066. doi: 10.1001/jamaoncol.2023.1629 37289451 10.1001/jamaoncol.2023.1629 PMC10251241

[50] M. Durante , R. Orecchia , J. S Loeffler . Charged‐particle therapy in cancer: clinical uses and future perspectives. Nat Rev Clin Oncol. 2017;14(8):483‐495. doi: 10.1038/nrclinonc.2017.30 28290489 10.1038/nrclinonc.2017.30

[51] G. A Viani , A. C Hamamura , C. V Arruda , C. E Cardoso , H. A Salmon , G. O Amaral . Children's outcomes in medulloblastoma proton versus photon craniospinal radiotherapy (CURE): meta‐analysis. Radiother Oncol. 2026;11:111367. doi: 10.1016/j.radonc.2026.111367 10.1016/j.radonc.2026.111367 41529731

[52] J. T. Yang , D. Yerramilli , E. Pentsova , et al. Proton craniospinal irradiation for patients with leptomeningeal metastasis. JAMA Oncol. 2025;11(11):1293. doi: 10.1001/jamaoncol.2025.3007 40906462 10.1001/jamaoncol.2025.3007 PMC12412039

[53] S. M. MacDonald , S. Pugh , R. Paulus , et al. Phase III randomized trial of proton vs. photon therapy for patients with non‐metastatic breast cancer receiving comprehensive nodal radiation: a radiotherapy comparative effectiveness (Radcomp) consortium trial: health‐related quality of life outcomes. Int J Radiat Oncol Biol Phys. 2025;123(4):1195. doi: 10.1016/j.ijrobp.2025.08.025

[54] S. J. Frank , P. M. Busse , J. J. Lee , et al. Proton versus photon radiotherapy for patients with oropharyngeal cancer in the USA: a multicentre, randomised, open‐label, non‐inferiority phase 3 trial. Lancet. 2026;407(10524):174‐184. doi: 10.1016/S0140‐6736(25)01962‐2 41391462 10.1016/S0140-6736(25)01962-2 PMC12812248

[55] C. B. Simone , J. P. Plastaras , S. K. Jabbour , et al. Proton reirradiation: expert recommendations for reducing toxicities and offering new chances of cure in patients with challenging recurrence malignancies. Semin Radiat Oncol. 2020;30(3):253‐261. doi: 10.1016/j.semradonc.2020.02.007 32503791 10.1016/j.semradonc.2020.02.007 PMC10870390

[56] M. Durante . Kaplan lecture 2023: lymphopenia in particle therapy. Int J Radiat Biol. 2024;100(5):669‐677. doi: 10.1080/09553002.2024.2324472 38442137 10.1080/09553002.2024.2324472

[57] M. Durante , J. Debus , J. S Loeffler . Physics and biomedical challenges of cancer therapy with accelerated heavy ions. Nat Rev Phys. 2021;3(12):777‐790. doi: 10.1038/s42254‐021‐00368‐5 34870097 10.1038/s42254-021-00368-5 PMC7612063

[58] B. R. Smith , J. St‐Aubin , D. E. Hyer . Commissioning of a motion management system for a 1.5T Elekta Unity MR‐Linac: a single institution experience. Appl. Clin. Med. Phys. 2025;26(4):e70005. doi: 10.1002/acm2.70005 10.1002/acm2.70005 PMC11969090 39955657

[59] A. U. Kishan , T. M. Ma , J. M. Lamb , et al. Magnetic resonance imaging–guided vs computed tomography–guided stereotactic body radiotherapy for prostate cancer. JAMA Oncol. 2023;9(3):365. doi: 10.1001/jamaoncol.2022.6558 36633877 10.1001/jamaoncol.2022.6558 PMC9857817

[60] S. M Bentzen , V. Gregoire . Molecular imaging–based dose painting: a novel paradigm for radiation therapy prescription. Semin Radiat Oncol. 2011;21(2):101‐110. doi: 10.1016/j.semradonc.2010.10.001 21356478 10.1016/j.semradonc.2010.10.001 PMC3052283

[61] K. Jasper , B. Stiles , F. McDonald , D. A Palma . Practical management of oligometastatic non–small‐cell lung cancer. J Clin Oncol. 2022;40(6):635‐641. doi: 10.1200/JCO.21.01719 34985915 10.1200/JCO.21.01719

[62] R. Mohan . A review of proton therapy—Current status and future directions. Precision Radiat. Oncol. 2022;6(2):164‐176. doi: 10.1002/pro6.1149 10.1002/pro6.1149 PMC9499036 36160180

[63] J. A. Efstathiou , B. Y. Yeap , J. M. Michalski , et al. Prostate advanced radiation technologies investigating quality of life (PARTIQoL): phase III randomized clinical trial of proton therapy vs. IMRT for localized prostate cancer. Int J Radiat Oncol Biol Phys. 2024;120(2):S1. doi: 10.1016/j.ijrobp.2024.08.012

[64] D. J. Thomson , J. M. Price , M. Tyler , et al. Proton beam therapy for oropharyngeal cancer (TORPEdO): a phase 3, randomised controlled trial. Lancet. 2026;407(10535):1259‐1275. doi: 10.1016/S0140‐6736(26)00314‐4 41875914 10.1016/S0140-6736(26)00314-4

[65] S.‐J. Lin , Q.‐J. Guo , Q. Liu , et al. International consensus guideline on delineation of the clinical target volumes at different dose levels for nasopharyngeal carcinoma (2024 version). Int J Radiat Oncol Biol Phys. 2025;123(2):415‐431. doi: 10.1016/j.ijrobp.2025.05.019 40419028 10.1016/j.ijrobp.2025.05.019

[66] F. B Geara , L. J. Peters , K. K. Ang , et al. Intrinsic radiosensitivity of normal human fibroblasts and lymphocytes after high‐ and low‐dose‐rate irradiation. Cancer Res. 1992;52(22):6348‐6352. 1423281

[67] N. Ghaderi , J. Jung , S. C Brüningk , A. Subramanian , L. Nassour , J. Peacock . A century of fractionated radiotherapy: how mathematical oncology can break the rules. Int J Mol Sci. 2022;23(3):1316. doi: 10.3390/ijms23031316 35163240 10.3390/ijms23031316 PMC8836217

[68] B. Venkatesulu , P. Giridhar , L. Pujari , et al. Lymphocyte sparing normal tissue effects in the clinic (LymphoTEC): a systematic review of dose constraint considerations to mitigate radiation‐related lymphopenia in the era of immunotherapy. Radiother Oncol. 2022;177:81‐94. doi: 10.1016/j.radonc.2022.10.019 36334694 10.1016/j.radonc.2022.10.019

[69] Z. Nowicka , K. Kuna , M. Łaszczych , K. Stawiski , B. Tomasik , W. Fendler . Effect of the time‐of‐day of radiotherapy administration on radiation‐induced lymphocyte depletion in lung cancer. Int J Radiat Oncol Biol Phys. 2024;120(2):e52. doi: 10.1016/j.ijrobp.2024.07.1892

[70] L. Cella , S. Monti , R. Pacelli , G. Palma . Modeling frameworks for radiation induced lymphopenia: a critical review. Radiother Oncol. 2024;190:110041. doi: 10.1016/j.radonc.2023.110041 38042499 10.1016/j.radonc.2023.110041

[71] L. Habimana‐Griffin , J. Prusa , B. Wang , et al. A novel focal duodenal radiation injury model reveals dose‐, time‐, and spatially dependent microbiome perturbations after radiation injury. Int J Radiat Oncol Biol Phys. Forthcoming 2025; 125: 590‐602. doi: 10.1016/j.ijrobp.2025.11.011 41248758 10.1016/j.ijrobp.2025.11.011 PMC13252817

[72] L. Galluzzi , M. J Aryankalayil , C. N Coleman , S. C Formenti . Emerging evidence for adapting radiotherapy to immunotherapy. Nat Rev Clin Oncol. 2023;20(8):543‐557. doi: 10.1038/s41571‐023‐00782‐x 37280366 10.1038/s41571-023-00782-x

[73] M. Durante , J. Flanz . Charged particle beams to cure cancer: strengths and challenges. Semin Oncol. 2019;46(3):219‐225. doi: 10.1053/j.seminoncol.2019.07.007 31451308 10.1053/j.seminoncol.2019.07.007

[74] M. Durante , J. Debus . Heavy charged particles: does improved precision and higher biological effectiveness translate to better outcome in patients?. Semin Radiat Oncol. 2018;28(2):160‐167. doi: 10.1016/j.semradonc.2017.11.004

[75] K. K Brock . Adaptive radiotherapy: moving into the future. Semin Radiat Oncol. 2019;29(3):181‐184. doi: 10.1016/j.semradonc.2019.02.011 31027635 10.1016/j.semradonc.2019.02.011 PMC7219982

